# Supplementary material for: Learning curve and surgical outcome of robotic assisted colorectal surgery with ERAS program
Source: Sci Rep. 2022 Nov 29;12:20566. doi: 10.1038/s41598-022-24665-w (PMC9709162; doi:10.1038/s41598-022-24665-w)
Supplement: Supplementary file 1 — Supplementary Information 1. [file 41598_2022_24665_MOESM1_ESM.zip › LSC Raw data-Ñ[▒K/099/H_ñw╝╨░O▒Kñσ.pdf]

正式摘要(電子簽章：000000201748748)		
W52 / W52 ☐英文標題(注意:無英文姓名-請補病人基本檔!)	☐【讀取】憑證	

臺中榮民總醫院
出院病歷摘要

轉入號 : 轉出號 :

[redacted]

**(8)**流水編號	
**(9)**入院日期	109/10/21 (2020) 直腸外科 W52 - 112
**(10)**轉科日期	
**(11)**出院日期	109/10/26 (2020) 住院天數計 5 日

診斷

- 入院 :Malignant tumor of sigmoid colon
- 出院 :Malignant tumor of sigmoid colon, s/p laparoscopic anterior resection and anastomosis on 10/22

主訴

- Stool OB+ while health examination 3 weeks ago

病史

- This 78 y/o has past history of
  1. CAD, s/p stent *5 in 中國 Hospital 20 years ago
  2. Gout at left heel, under medication control
  This time, the patient got stool OB+ while health examination. He denied diarrhea, constipation, abdominal fullness nor discomfort recently. No alcohol drinking, smoking nor betel nut chewing history. No allergy nor family history. CT scan showed sigmoid cancer without regional lymphadenopathy or intra-abdominal metastasis, Under the impression of sigmoid colon cancer, he was admitted to our ward for laparoscopic Anterior resection and anastomosis.

體檢發現

-
  - General appearance :
    - easy looking , conscious clear , without cardiopulmonary distress
    - Vital signs自動帶入date 體溫(BT) 脈搏(P) 呼吸(R) 血壓(BP) 身高(BH) 體重(BW) BMI
      20201021 165.9cm 80.3Kg 29.2
    - Vital signs: B.P. 125/55 mmHg , P.R. 59 /min , R.R. 19 /min , B.T. 36.8 ℃
  - HEENT :
    - Eyes: no anemic conjunctiva, no icteric sclera, normal EOM
    - ENT: no otorrhea, no rhinorrhea, no erythematous mucosa, no injected tonsil, no deformity
  - Neck: supple, freely movable, trachea midline,no deformity, no engorged jugular vein , no palpable thyroid or lymph node enlargement
  - Chest:
    - Inspection: Grossly normal, symmetric and fully expansion, no accessory muscle used, no operation scar, no spider angiomata, no gynecomastia
    - Palpation: no palpable mass or nodule, no subcutaneous emphysema, no tenderness
    - Ausculation: clear breathing sound
  - Heart:
    - Palpation: no thrill, no heave
    - Percussion: normal heart size
    - Ausculation: regular heart beat, normal S1, S2, no gallop, no murmur
  - Extremities: No deformity, full ranged of motion without limitation, no clubbing finger or nail cyanosis, no pitting edema, no ulcer or pigmentation, no obvious skin lesion, no varicose vein, no bone, joint or muscle tenderness

CRS CONDITION

- Karnofsky performance status: (100) Normal, no complaints, no evidence of disease
- PE of Abdomen:
- CEA: 1.38 ng/ml
- Imaging Studies:
- Colonoscope report: .
- Figures:
  1. colon & rectum

手術日期及方法

- 手術日期及方法20201022 Laparoscopic Anterior resection and anastomosis - malignant

住院治療經過

- After admission, pre-op survey was done and no significant abnormality was noted. Laparoscopic anterior resection and anastomosis was executed smoothly on 10/22. After the surgery, the patient follow ERAS protocol and recovered gradually. Under stable condition, the patient was discharged and OPD f/u was arranged.

合併症

一般檢查

- 累積報告：

DATE	TIME	ALB	TP	BIL.T	BIL.D	ALKP	AST	ALT	LDH	CHOL	TG	HDL-C	LDL	UA	NH3	AMY
1091021	1712		7.0							146	112		66	4.6		
1091022	2035	3.7		0.8	0.2	51	19	20								

DATE	TIME	WBC	RBC	HGB	HCT	MCV	PLT	BLST	NEUT	BAND	SEG	LYM	MON	EOS	BAS
1091021	1711	8470		12.3			172		59.9			34.4	5.1	0.5	0.1
1091022	2035	10910	4.03	12.5			161		88.3			10.4	1.1	0.0	0.2

DATE	TIME	NA	K	CL	CA	BUN	CREAT	Mg	P	CRP	ESR	PCT	LIPASE	LACTATE
1091021	1712	139	4.2			21	1.21							
1091022	2035	140	4.0	107	8.3	16	1.04							
1091026	0927	139	3.7		7.7	10	1.01	2.1	2.4					

DATE	TIME	GLUCOSE	PAB	TRF	ZN	CU	KETONE	CK	CKMB	PT-P	PT-C	APTT-P	APTT-C	TRO-T
1091021	1711									11.1	10.3	29.8		
1091022	2035	130												

特殊檢查

病理報告

其它

出院時情況

- 3.改門診治療

出院指示

藥名	劑量	劑量單位	用法	頻次	數量	數量單位	現況	天數	單位
OCAS Harnalidge 0.4 mg	1	TAB	PO	QDAC	7	TAB	TH	7	天
Lactam tab 500mg	1	TAB	PO	Q6HPRN	20	TAB	TH	5	天

出院計劃

主治醫師 2062F 蔣鋒帆 / 住院醫師 9972C 陳智翔

正式摘要(電子簽章：000000201748748)

W52 / W52

doctorRank:B
